# Estimating the impact of violent events on transmission in Ebola virus disease outbreak, Democratic Republic of the Congo, 2018–2019

**DOI:** 10.1016/j.epidem.2019.100353

**Published:** 2019-07-26

**Authors:** S. Rae Wannier, Lee Worden, Nicole A. Hoff, Eduardo Amezcua, Bernice Selo, Cyrus Sinai, Mathias Mossoko, Bathe Njoloko, Emile Okitolonda-Wemakoy, Placide Mbala-Kingebeni, Steve Ahuka-Mundeke, Jean Jacques Muyembe-Tamfum, Eugene T. Richardson, George W. Rutherford, James H Jones, Thomas M. Lietman, Anne W. Rimoin, Travis C. Porco, J. Daniel Kelly

**Affiliations:** a Francis I. Proctor Foundation for Research in Ophthalmology, San Francisco, University of California, CA, USA; b Department of Epidemiology and Biostatistics, School of Medicine, University of California, San Francisco, San Francisco, CA, USA; c Department of Epidemiology, School of Public Health University of California, Los Angeles, CA, USA; d Ministry of Health, Kinshasa, Democratic Republic of Congo; e Department of Geography at University of North Carolina, Chapel Hill, NC, USA; f School of Public Health, University of Kinshasa, Kinshasa, Democratic Republic of Congo; g Insitut National de Recherche Biomedicale, Kinshasa, Democratic Republic of Congo; h Global Health and Social Medicine, Harvard Medical School, MA, USA; i Department of Earth System Science, Stanford University, Stanford, CA, USA; j Woods Institute for the Environment, Stanford University, Stanford, CA, USA; k Department of Ophthalmology, School of Medicine, University of California, San Francisco, San Francisco, CA, USA

**Keywords:** Ebola virus disease, Outbreak, Mathematical modeling, Geospatial, Democratic Republic of Congo, Africa

## Abstract

**Introduction:**

As of April 2019, the current Ebola virus disease (EVD) outbreak in the Democratic Republic of the Congo (DRC) is occurring in a longstanding conflict zone and has become the second largest EVD outbreak in history. It is suspected that after violent events occur, EVD transmission will increase; however, empirical studies to understand the impact of violence on transmission are lacking. Here, we use spatial and temporal trends of EVD case counts to compare transmission rates between health zones that have versus have not experienced recent violent events during the outbreak.

**Methods:**

We collected daily EVD case counts from DRC Ministry of Health. A time-varying indicator of recent violence in each health zone was derived from events documented in the WHO situation reports. We used the Wallinga-Teunis technique to estimate the reproduction number *R* for each case by day per zone in the 2018–2019 outbreak. We fit an exponentially decaying curve to estimates of *R* overall and by health zone, for comparison to past outbreaks.

**Results:**

As of 16 April 2019, the mean overall *R* for the entire outbreak was 1.11. We found evidence of an increase in the estimated transmission rates in health zones with recently reported violent events versus those without (*p* = 0.008). The average *R* was estimated as between 0.61 and 0.86 in regions not affected by recent violent events, and between 1.01 and 1.07 in zones affected by violent events within the last 21 days, leading to an increase in *R* between 0.17 and 0.53. Within zones with recent violent events, the mean estimated quenching rate was lower than for all past outbreaks except the 2013–2016 West African outbreak.

**Conclusion:**

The difference in the estimated transmission rates between zones affected by recent violent events suggests that violent events are contributing to increased transmission and the ongoing nature of this outbreak.

## 1. Introduction

Since 1976, 10 of the over 34 reported Ebola virus disease (EVD) outbreaks have been in the Democratic Republic of the Congo (DRC) (World Health Organization Regional Office for Africa, 2018; Anon, 2019). The current 2018–2019 EVD outbreak in northeastern DRC is, as of April 2019, the second largest EVD outbreak in history and the first to occur in a conflict setting (World Health Organization Regional Office for Africa, 2019). Although its magnitude substantially trails the 2013–2016 EVD outbreak in West Africa, the current outbreak has surpassed epidemic forecasts, particularly mathematical modelling studies that used historical data from prior outbreaks (Worden et al., 2018; Kelly et al., 2019; Enanoria et al., 2015; Ebola haemorrhagic fever in Sudan, 1978; Breman et al., 2016). These studies had projected a final outbreak size of up to 1295 cases as of 25 February 2019 while the current outbreak now has reported 1604 cases (Worden et al., 2018; Kelly et al., 2019).

Since the EVD outbreak began, there have been reports of violent events that have ranged from destruction of Ebola care facilities and injured and murdered healthcare workers to events unrelated to Ebola care, linked to elections and local unrest (World Health Organization Regional Office for Africa, 2019; Christopher Dickey, 2018; Branswell, 2018, 2019a, 2019b, 2019c, 2019d). Following many of these violent events, Ebola response activities have been disrupted. In some cases, new EVD case counts have increased in the district affected following these events, despite intensive public health interventions comprising the deployment of rapid diagnostic tests, novel therapeutics, contact tracing, and ring vaccination using a vaccine approved for emergency use with an estimated 97.5% efficacy (ElSherif et al., 2017; World Health Organization Regional Office for Africa, 2019; WHO, 2019). There has been a growing sentiment that such events may be contributing to EVD transmission, but quantitative analysis is lacking.

Here, we hypothesized that during the current outbreak to date, there had been higher transmission rates in zones that had recently experienced violent events than in zones that had not experienced such events. Furthermore, we also hypothesized that Shannon entropy computed with respect to space, a measure of the spatial spread of cases and the uniformity of their distribution across districts in this epidemic, has increased over time.

## 2. Methods

### 2.1. Data

A time series of case counts were collected from situation reports presented by the DRC Ministry of Health and were confirmed using situation reports posted by the World Health Organization (WHO) (World Health Organization Regional Office for Africa, 2019). EVD cases were classified as suspected, probable, or confirmed. Suspected cases underwent diagnostic testing and were subsequently classified either as confirmed or not confirmed. Cases reported post-mortem were classified as probable based upon their epidemiological history. The symptom onset date and the reporting date of probable, confirmed, and suspected cases were collected from the beginning of the outbreak on 8 May 2018 through 15 April 2019. Beginning on 8 August 2018, data by health zone were also collected for probable and confirmed cases. As the outbreak has continued, cases were reassigned by the DRC Ministry of Health from their initially reported health zone to the health zone where epidemiological evidence pointed to EVD acquisition. Symptom onset data were later made available going back to the beginning of the outbreak.

Given the dynamic nature of violence in the region and the relatively short generation time of EVD, we undertook a time-varying, health zone-level analysis of the outbreak comparing transmission in zones that had experienced recent violence to those without recently reported violence. We considered a violent event as one reported within the WHO situation reports until 15 April 2019 (World Health Organization Regional Office for Africa, 2019). We assigned each health zone as having been exposed to violent events or not. After a violent event, we considered the following three weeks as the exposure period. We modeled the effect of violent events within the previous week, two weeks, three weeks, four weeks, and five weeks to determine the sensitivity to the time period chosen.

### 2.2. Statistical analysis

We used the Wallinga-Teunis technique to estimate the number of secondary cases *R* for each case in the 2018–2019 outbreak (Wallinga and Teunis, 2004). We defined the serial interval as the interval between disease onset in an index case and disease onset in a person infected by that index case. We used both symptom onset and case report data in our analyses given the limitations of each dataset. Historical outbreaks used case report data, though case report data in this dataset is subject to case reclassification between zones, reflecting epidemiologic knowledge on the place of transmission rather than place of report, that can show up as transmission. In the current outbreak, case report data were updated nearly daily from the official daily totals reported by the DRC Ministry of Health and revised as needed. Thus, while the symptom onset data may be the fundamental definition of the serial interval, these data were not always revised as more accurate information became available. We employed a gamma distribution with a mean of 14.5 days and a standard deviation of 5 days for the serial interval distribution. This models a serial interval of EVD cases approximately ranging from 3 to 36 days, with mean 14 to 15 days (Aylward et al., 2014; Wong et al., 2017; Chowell and Nishiura, 2014). Our application of the Wallinga-Teunis method assumes there are no missing cases.

To compare transmission in the current outbreak to past outbreaks, we applied the same estimation technique to reported cases by date from 18 prior outbreaks (Ebola haemorrhagic fever in Sudan, 1978; Breman et al., 2016; Baron et al., 1983; Georges et al., 1999; Khan et al., 1999; CDC, 2001; Anon, 2003; Boumandouki et al., 2005; World Health Organization Abonnement Annuel, 2005; Nkoghe et al., 2011; Rosello et al., 2015). We estimated the initial reproduction number *R*_initial_ and exponential decay (or quenching) rate τ for each outbreak by fitting an exponentially decaying curve *R*(*d*) = *R*_initial_*e*^−τ*d*^ to the outbreak’s estimates of *R* by day *d*. This exponential decay “quenching” parameter approximates the often observed reduction in *R* over the course of an outbreak that may be due to phenomena such as formal control efforts including case finding and quarantine as well as less formal responses including individual behavioral changes or local depletion of susceptibles. This equation approximates temporal change in transmission rate by a smoothed quenching pattern in which *R* decreases exponentially over time at a rate defined by the quenching parameter. The estimates *R*_initial_ and τ obtained by this fit are reported for comparison to historic outbreaks and not used for further modeling.

We repeated the Wallinga-Teunis procedure to estimate the reproduction number *R* separately for each health zone, creating a time series of estimated reproduction numbers for each zone. When estimating *R* for each zone, we considered the possibility of transmission between regions. The probability of an inter-zone transmission relative to intra-zone transmission is denoted *ω*, representing the reduced probability of a case transmitting outside of its health zone as compared to their probability of transmitting within their own health zone. We compared estimates based on values of the inter-zone transmission-mixing parameter *ω* across its range from 0 (no transmission allowed between regions) to 1 (all cases in all regions transmit equally to all other cases, regardless of region).

Confidence intervals for the Wallinga-Teunis estimated *R* by health zone were simulated using the calculated probabilities for each case *i* transmitting to case *j* to probabilistically assign a transmission link for every case, based on 5000 simulations (Wallinga and Teunis, 2004). Using the distribution of *R* per day per zone, we then calculated the negative-binomial confidence interval for each day per zone.

Statistical inference on the resulting estimates of *R* by day per zone was conducted by regressing estimated *R* on the presence of a recently reported violent events (Bartlett, 1946; Politis, 2003). To adjust for autocorrelation, the standard errors of the estimates were estimated using a time-series bootstrap with blocks of 7 days over 2048 replications.

The Shannon entropy of the cumulative number of cases was computed based on all health zones and standard errors were computed from the proportion in each geographic region (From, 2003). Time series bootstrap based on a fixed length of 7 was used to compute standard errors of the time trend, based on ordinary least squares linear regression of entropy on days since the first case.

We conducted all analyses in R (v. 3.5 for Macintosh, R Foundation for Statistical Computing, Vienna, Austria).

## 3. Results

**Fig. 1.**
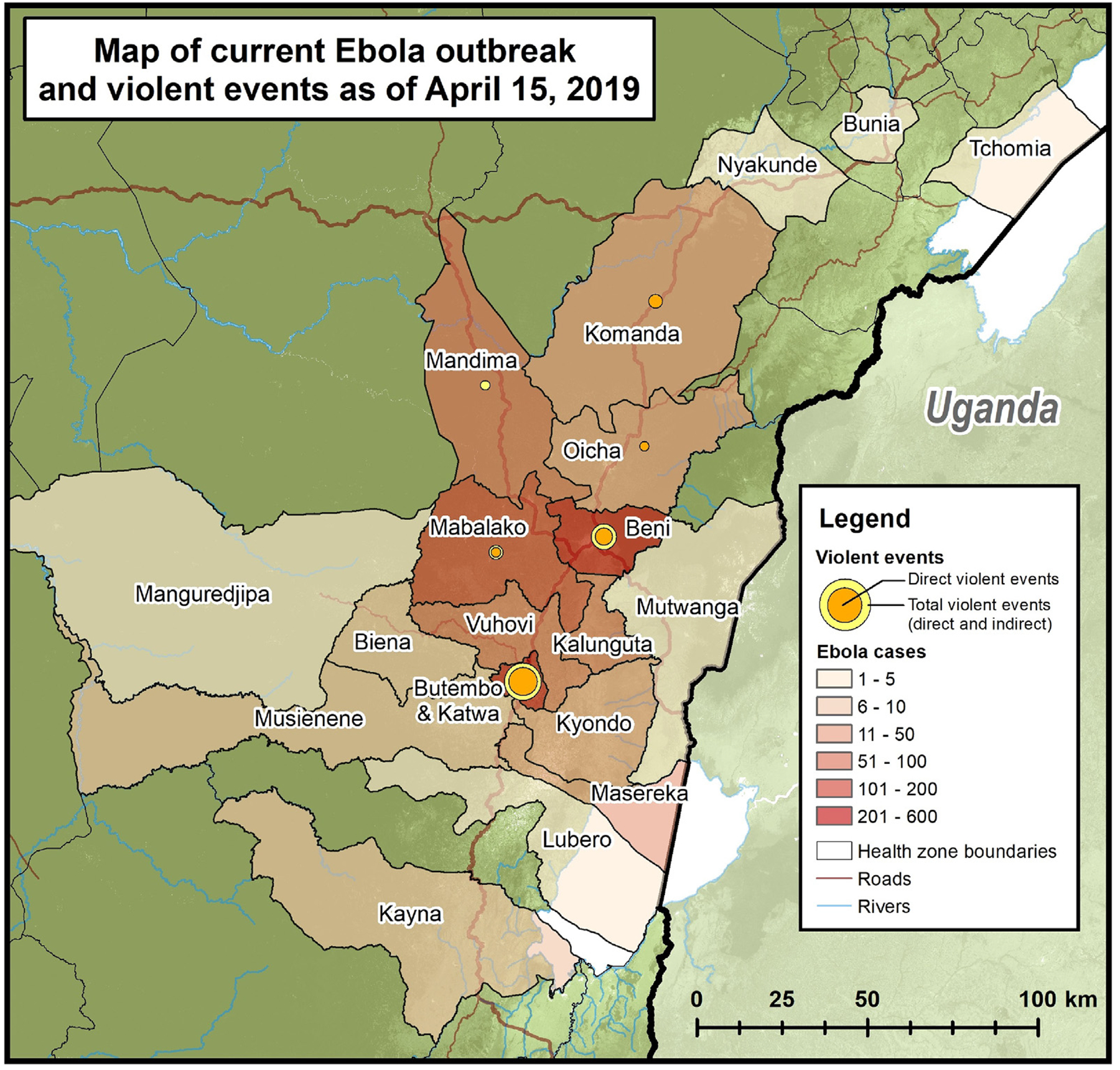
Map of current Ebola outbreak and violent events. The 2018–2019 EVD outbreak in northeastern DRC as of 15 April 2019 with confirmed and probable EVD cases depicted by health zone by color. Violent events are represented as inner circle = direct events where violence was directed at Ebola relief efforts; outer circle = all events where violence either indirectly or directly impacted Ebola relief activities. (For interpretation of the references to color in this figure legend, the reader is referred to the web version of this article.)

As of 15 April 2019, a total of 1273 EVD cases had been reported. Thus far during the outbreak period, a total of 1044 cases have been reported in the seven health zones that have experienced violent events whereas 229 cases have been reported in zones without violent events. The outbreak has been centered in health zones of Katwa (32%), Beni (20%), Mabalako (8%), Butembo (9%). Butembo and Beni are the health zones with the most violent events (Fig. 1).

Since the beginning of the outbreak in May 2018, the average reproduction number (mean *R*_estimate_) was 1.11. Using case report data, after August 8, 2018, the average reproduction number was between 1.12 and 1.23 in the 21 days following a violent event in a district, and between 0.81 and 1.08 in all other cases (Fig. 3). The difference between reproduction numbers with and without violent events was statistically significant and increased as the mixing between zones was assumed increasingly limited (transmission mixing parameter *ω* decreased). Even at very high levels of inter-zone transmission mixing, corresponding to relatively homogenous transmission within and across health zones, (*ω* = 0.8, *p* = 0.016), the difference was still statistically significant (Fig. 3).

**Fig. 2.**
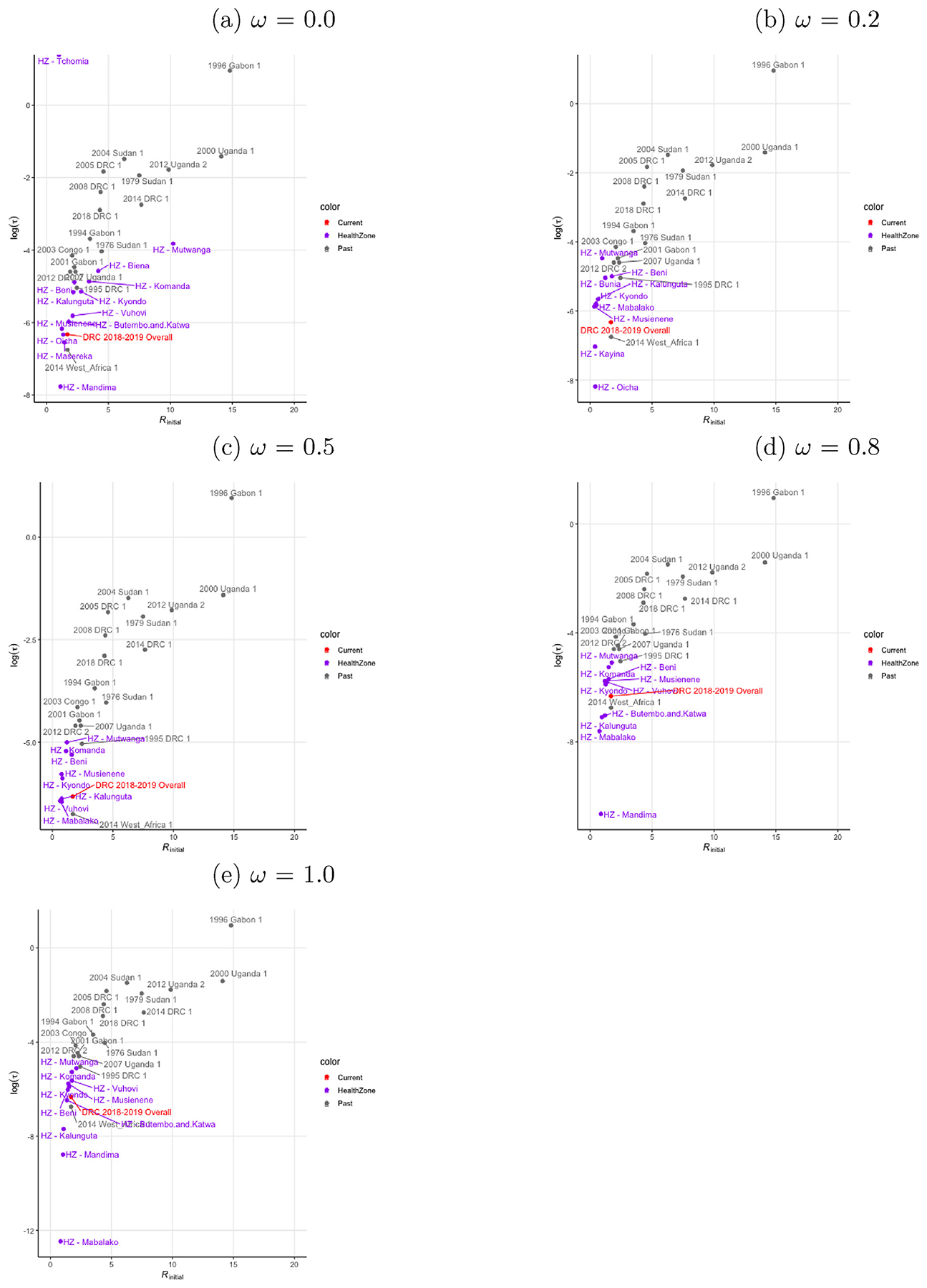
Estimated initial reproduction number *R*_initial_ and quenching rate τ for current and past outbreaks. *R*_initial_ and τ were estimated for the current outbreak as an overall summary measure, as well as independently in health zones with over a range of inter-zone transmission mixing parameters *ω*. (a) No transmission between zones: *ω* = 0.0. (b) Low transmission between zones: *ω* = 0.2. (c) Medium transmission between zones: *ω* = 0.5. (d) High transmission between zones: *ω* = 0.8. (e) Full transmission between zones: *ω* = 1.0.

**Fig. 3.**
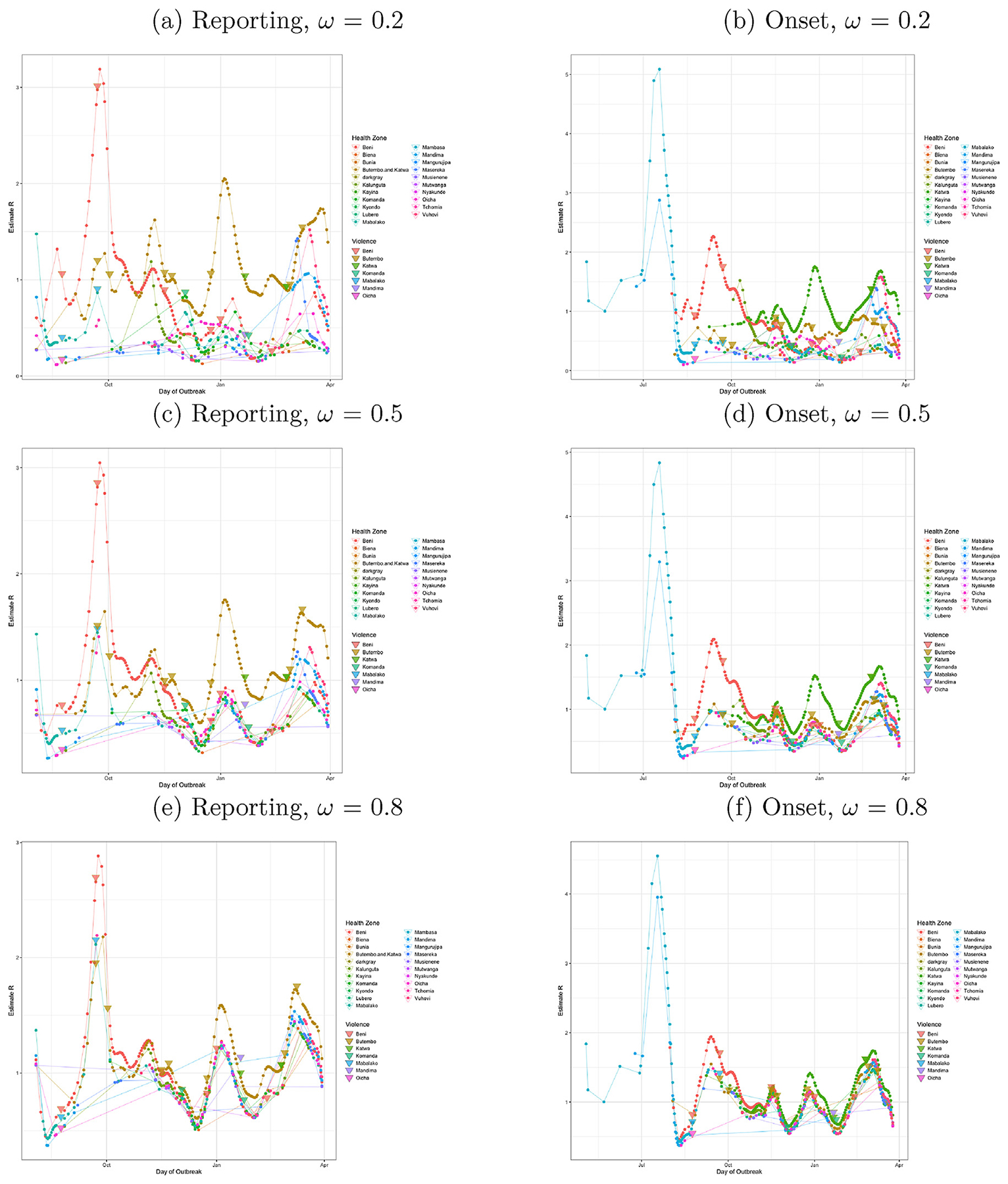
Wallinga-Teunis estimated *R* per day and violent events by health zone for symptom onset and reporting dates, allowing for mixing between regions. *R* was estimated over a range of inter-zone transmission mixing parameters *ω*. Violent events are marked using triangles with colors matching the affected district(s). (a, b) Low transmission between zones: *ω* = 0.2. (c, d) Medium transmission between zones: *ω* = 0.5. (e, f) High transmission between zones: *ω* = 0.8.

We compared the initial estimated *R* and the quenching parameters of past outbreak to the current outbreak and its respective health zones (Fig. 2). The inter-zone transmission mixing parameter *ω* was varied over a range from 0 to 1 (Fig. 2). The log of the estimated quenching parameter was (−6.32) for the current outbreak was closest, though slightly higher, to that estimated for the 2013–2016 outbreak in West Africa (−6.87). This was paired with a slightly higher estimated *R*_initial_ = 1.69 than for the West African outbreak where *R*_initial_ = 1.67. The (*R*_initial_) reported here for the West African outbreak is consistent with the previous literature (Barbarossa et al., 2015; Lau et al., 2017). These numbers are consistent with the long trajectory and continued transmission of the outbreak as a whole, although the trend of observed *R* in the current outbreak is rather different from the West African outbreak where the mean overall *R* was only above 1.0 early in the outbreak before dropping below 1.0 as the outbreak continued, where here it can be seen to fluctuate with three peaks above *R*_estimate_ = 1.5 and below *R*_estimate_ = 0.5 (Fig. 3) (Lau et al., 2017). The estimated *R*_initial_ and quenching parameters clustered around the current outbreak estimates, some more extreme (lower quenching) and other more like smaller outbreaks of the past. Each health zone was also estimated. Health zones at the center of the outbreak appeared on average to have lower quenching. Mabalako, Butembo, and Katwa, for example, often had weaker quenching than the West African outbreak; however, this was not consistent because Beni consistently reported a higher level of quenching from the estimates, indicating the complex geographic distribution of transmission among health zones.

In evaluating a probable mixing parameter to use for evaluating the results from this outbreak, we can look at the evidence from the time series of *R*_estimate_ by health zone and examine their behavior (Figure 3). Both extremes of no mixing (*ω* = 0.0) or full mixing (*ω* = 1.0) are unrealistic and lead to inconsistent results. When no mixing is allowed (*ω* = 0.0) this causes false spikes in *R*_estimate_ in health zones with low levels of transmission following an increase in transmission in a neighboring health zone as the neighboring cases are unable to account for the spread of transmission to the low transmission zone, and this creates a false apparent spike in their own health zone to compensate for a lack of inter-zone transmission. Even low levels of mixing (*ω* = 0.2) are enough to remove false spikes in *R*_estimate_ that we see when no mixing is allowed (*ω* = 0.0). When full mixing is allowed (*ω* = 1.0) this leads to all health zones having identical *R*_estimate_ at each time point. This herding behavior is strongly seen still when (*ω* = 0.8) at unrealistically high levels. Even at a middle level of mixing (*ω* = 0.5) it appears that health zones herd too strongly to the health zone driving transmission, as spikes in transmission at days 200, 235 and 304 cause all health zones reporting cases to spike as well, when it is most probable the increase in transmission is being driven only by a few health zones and the others only responding to the initial change in transmission. Although we do not make an effort to formally estimate a probable mixing parameter, it would be reasonable to consider the estimates taken with *ω* = 0.1 to *ω* = 0.5 as these are the probable limits of the range for the mixing parameter in this outbreak.

**Fig. 4.**
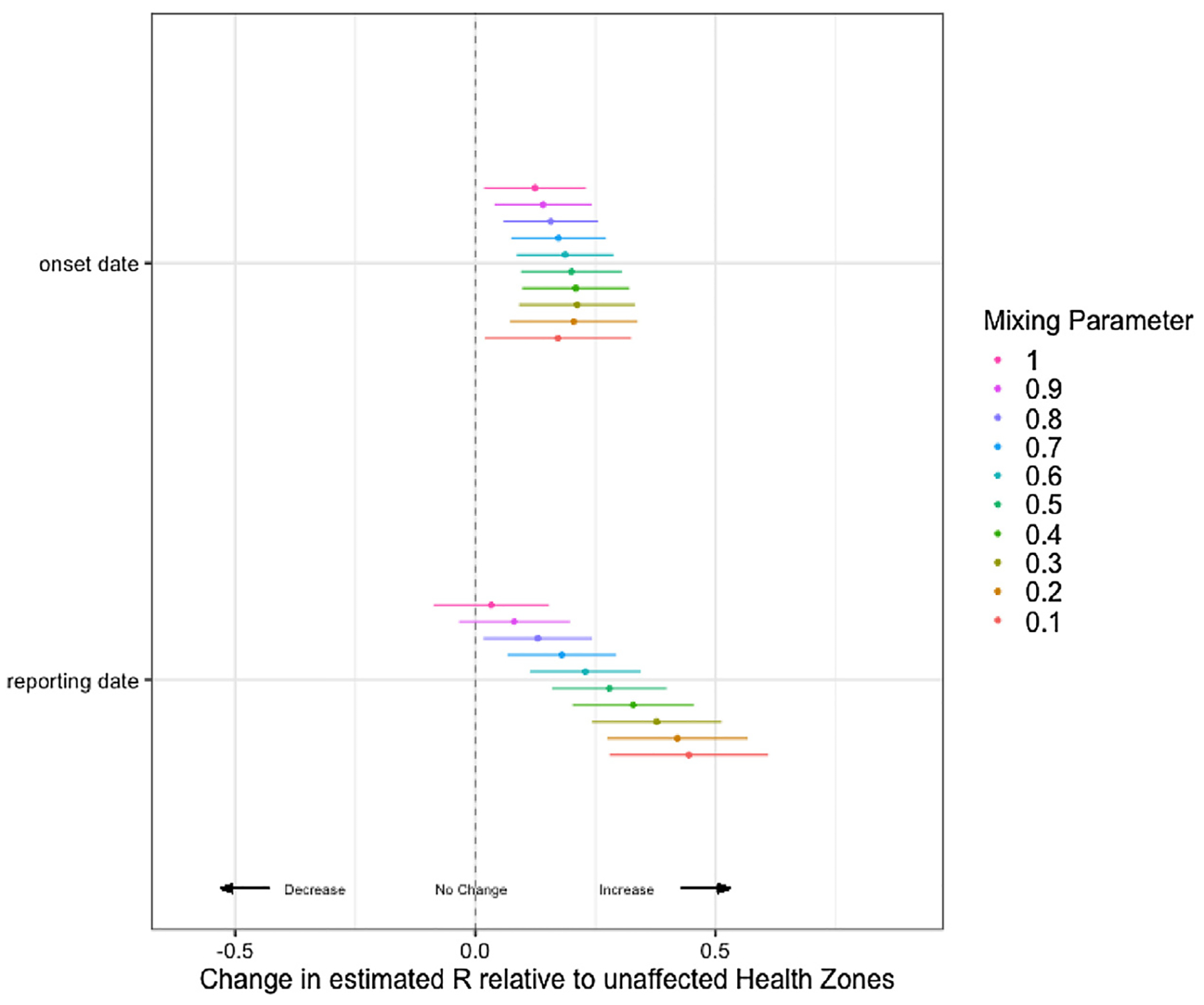
Regression estimates of the effect of violent events within the last 21 days on *R*_est_ using case report and symptom onset date data over a range of inter-zone transmission mixing parameters *ω*, where *ω* = 0.0 no mixing between zones and *ω* = 1.0 full transmission between zones.

**Fig. 5.**
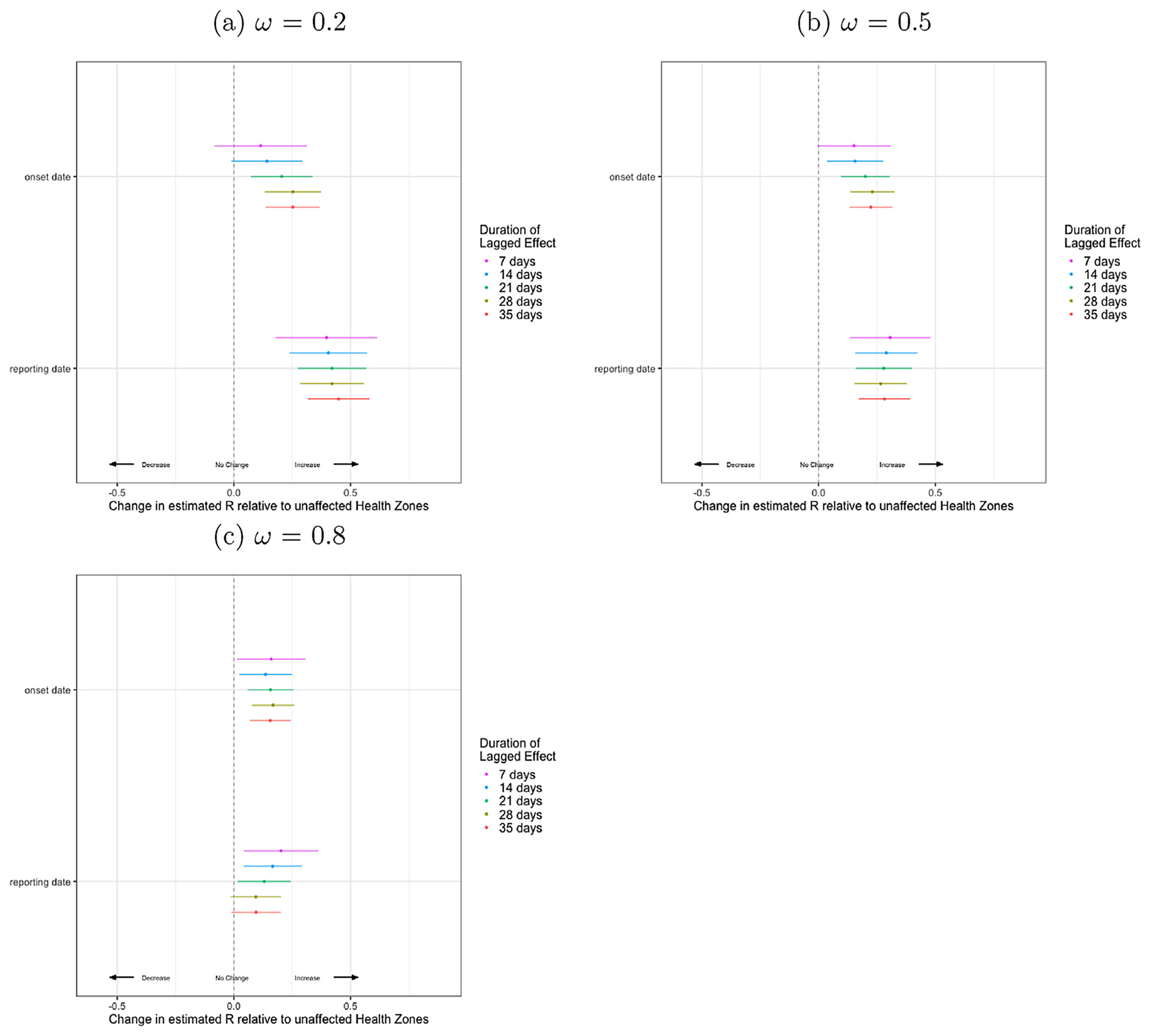
Wallinga-Teunis estimated *R* per day by health zone for symptom onset and reporting dates, Regression estimates of the effect of recent violent events upon *R*_est_ using case reporting and symptom onset date data over a range of inter-zone transmission mixing parameters *ω*, where *ω* = 0.0 no mixing between zones and *ω* = 1.0 full transmission between zones. (a, b) Low transmission between zones: *ω* = 0.2. (c, d) Medium transmission between zones: *ω* = 0.5. (e, f) High transmission between zones: *ω* = 0.8.

Using case report data, at *ω* = 0.1 to *ω* = 0.8 we see a difference in the *R*_estimate_ by violent events (Fig. 4). Looking only at *ω* between 0.1 and 0.5, we see an increase in *R*_estimate_ following violent events of 0.44 (95% CI: 0.28, 0.61, *p* < 0.001) to 0.28 (95% CI: 0.16, 0.40, *p* < 0.001). However, when considering the symptom onset data the strength of the effect of recently reported violence was reduced, though still significant overall with violent events leading to an increase in *R*_estimate_ of 0.17 (95% CI: 0.02, 0.32, *p* = 0.026) to 0.20 (95% CI: 0.10, 0.30, *p* < 0.001) (Fig. 5).

To assess the sensitivity of the lag time chosen after a violent event, we looked at the effect of violent events reported in increments of increasing weeks. Looking at the case report data with *ω* = 0.3, the overall effect size is fairly constant whether we consider a period of 7 days or 35 days, with an increase in *R*_estimate_ of 0.37 (95% CI: 0.17, 0.57, *p* < 0.001) and 0.40 (95% CI: 0.28, 0.52, *p* < 0.001), respectively.

When looking at the onset data with *ω* = 0.3, the strength of the effect of recent violent events is strongest when it is evaluated over a full 21–28 days, as *R*_estimate_ increases from 0.13 (95% CI: −0.05, 0.31, *p* = 0.074) to 0.21 (95% CI: 0.09, 0.33, *p* < 0.001) as the lag increases 7 to 21 days and then becomes relatively stable.

We did not consider event times longer than 35 days apart because many of the affected health zones have events that occur less than one month apart, sometimes occuring as little as one week apart. For these health zones, increasing duration beyond this point does not increase the period of time considered as being impacted by violent events, and thus we lose much of our ability to further distinguish between transmission in zones with recent violent events.

**Fig. 6.**
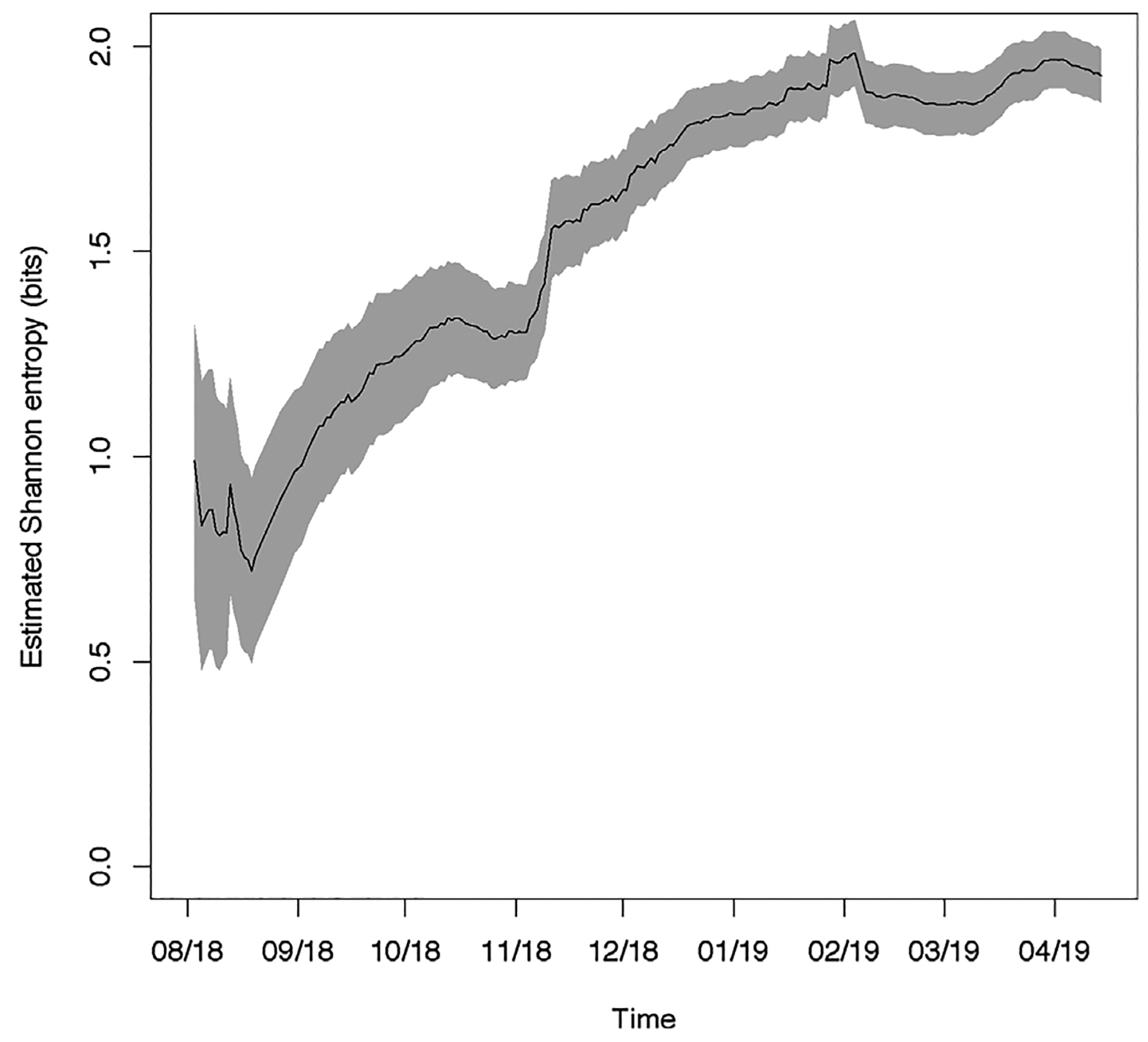
Estimated Shannon entropy of 2018–2019 DRC outbreak.

Fig. 6 shows the estimated Shannon entropy over the course of the epidemic. The estimated entropy was 0.99 ± 0.17 on 17 August 2018, rising to 1.88 ± 0.04 by 15 April 2019. We find evidence of an increasing trend (*p* < 0.01, time series bootstrap), showing less concentration of cases over time. Some increase in entropy is expected early in an epidemic, as cases begin to appear in new health zones. Later in an epidemic, a decrease in entropy could occur if there were an increased concentration of cases in a few of the affected health zones. It is possible that the continued spatial spread of the current outbreak, which is second only to the West African outbreak, is contributing to the difficulty in controlling this outbreak.

## 4. Discussion

Among health zones situated within the EVD outbreak in DRC, we found that the EVD transmission rate (reproduction number *R*) was higher following violent events. The outbreak was subcritical (*R* < 1.0, non-sustaining transmission) in zones without violent events reported by WHO (World Health Organization Regional Office for Africa, 2019), while it was supercritical (with estimated *R* > 1.0, continued transmission) in zones with reported violent events, suggesting that ongoing violence is likely perpetuating an otherwise declining outbreak.

Our findings suggest that violent events increased transmission in the weeks following a violent event, and that this effect may be sustained for many more weeks. After the destruction of the Ebola care facility in Katwa, for example, over one month was needed before Ebola virus disease relief efforts were fully resumed. Our time series regression found an effect of violent events on estimated *R* across both symptom onset data and case report data, across all plausible levels of inter-region transmission (Fig. 3), and lagged follow-up periods of 14 or more days. The consistency of the effect of recent violent events across data sets strongly supports the idea that violence is indeed contributing to the increased transmission and ongoing nature of this outbreak (Richardson and Fallah, 2019; Frankfurter et al., 2019; Richardson et al., 2016a, 2016b, 2017a, 2017b). More research is needed to confirm and further understand how the frequency and intensity of events may affect the contributions of violence to EVD transmission.

There are several limitations in our analysis. Cases may have escaped detection and reporting (Richardson et al., 2016a, 2016b; Kelly et al., 2018). If cases were missing in a biased way that systematically under-reported cases in certain areas or at certain times, there could have been a biased estimate of the effect, though the direction of the bias would be unclear. Additionally, if there was heterogeneity in the reporting delays, this could have biased the effect estimates made using the case report data, though this objection would not apply to the symptom onset data. The nature and causes of violent events can be quite different. Moreover, such events can affect different numbers of people, can vary in geographic scope as well as the duration of their impact. Other non-violent events not considered in this analysis, such as strikes, may also be contributing to ongoing transmission. Unmeasured causes or determinants of violence in the region may also drive variation in transmission or reporting in an unmeasured way. The epidemic curve in each health zone describes relatively few cases, making interpretations of specific features of their epidemic curves unwise. Our analysis relied on WHO reporting of violent events, and more accurate quantification of these events may be possible. Note that the assumption of exponential decay (quenching) of *R*_initial_ may not accurately characterize this epidemic; however, this was only used for comparisons to past outbreaks, and the estimates of the effect of violent events are not affected by this. While we have shown that the ongoing violence has likely hampered control and contributed to this becoming the second largest epidemic, our efforts to quantify the role of violence should be interpreted with caution. These considerations also suggest that meaningful interventions must consider the socio-political determinants of armed conflict in the DRC, including the legacies of colonialism, and other forms of historical and ongoing violence (Sweeney, 2019; Nzongola-Ntalaja, 2013; Council on Foreign Relations, 2019; Richardson and Fallah, 2019; Frankfurter et al., 2019; Richardson et al., 2016a, 2016b, 2017a, 2017b).

On 16 April 2019, the Democratic Republic of the Congo’s Minister of Health addressed the worsening outbreak and indicated that with “the difficult security situation, this epidemic had gone beyond public health” (Rdc, 2019). While this may have been the first time an EVD outbreak occurred in an active conflict zone, it is unlikely to be the last time that violent conflict contributes to the prevention of epidemic decline. As EVD surges in DRC, a polio outbreak surges in a conflict area of Nigeria. Ebola virus and other infectious disease outbreaks have the potential to become neglected crises in conflict settings if left unchecked. Despite immense security challenges, Ebola responders and the people in the affected area work tirelessly under dangerous conditions and deserve great respect, gratitude, and protection for their ongoing work to contain this public health disaster.
